# A Novel MATLAB‐Based Quantification of Microincision Cyclotorsion and Its Impact on Myopic Astigmatism Correction in KLEx

**DOI:** 10.1155/joph/7176960

**Published:** 2026-09-20

**Authors:** Jie Hou, Feng Rao, Yulin Lei, Jing Zhang, Yahui Dong

**Affiliations:** ^1^ Jinan Mingshui Eye Hospital, Jinan 250200, China; ^2^ School of Electrical and Information Engineering, Changzhou Institute of Technology, Changzhou 213002, China, czu.cn

**Keywords:** astigmatism, cyclotorsion, microincision, myopia, small incision lenticule extraction

## Abstract

**Purpose:**

To develop an automated image recognition method for the identification of corneal microincisions in keratorefractive lenticule extraction (KLEx) and assess microincisional cyclotorsion and its impact on postoperative astigmatism correction using vector analysis.

**Methods:**

This retrospective study included 92 patients (184 eyes) who underwent KLEx. Postoperative slit‐lamp images were analyzed using a custom MATLAB‐based program. The cyclotorsion angle and direction of the microincisions were quantified. Vector analysis was used to evaluate postoperative astigmatism correction at one week, one month, and three months. Correlations between cyclotorsion magnitude and preoperative ocular parameters were analyzed, and astigmatic outcomes were compared between low (≤ 4°) and high (> 4°) cyclotorsion groups.

**Results:**

The mean absolute microincision cyclotorsion angle was 4.19° ± 2.92° (range: 0°–12.43°) in right eyes and 4.53° ± 3.36° (range: 0°–13.74°) in left eyes, with no significant interocular difference (*p* > 0.05). Outward incision cyclotorsion was observed in > 80% of eyes. No significant correlations were found between the cyclotorsion magnitude and preoperative parameters, including age, spherical equivalent, central corneal thickness, keratometry, or *Q* value (all *p* > 0.05). At 3 months, there were no significant differences in vector parameters between low and high cyclotorsion groups, though the correction index tended to be slightly lower in the high‐cyclotorsion group (0.93° ± 0.32° vs. 1.04° ± 0.40°, *p* > 0.05).

**Conclusion:**

An automated MATLAB‐based method can be used to quantify the cyclotorsional movement of microincisions in KLEx. The magnitude of corneal microincisional cyclotorsion does not appear to have a significant effect on the efficacy of corneal astigmatism correction.

## 1. Introduction

Keratorefractive lenticule extraction (KLEx) is a standard refractive procedure used to treat myopia and myopic astigmatism. Although KLEx demonstrates excellent efficacy in correcting spherical refractive errors, its performance in astigmatic correction remains controversial [1, 2]. One major contributing factor to residual astigmatism is ocular cyclotorsion [3, 4].

Previous studies have demonstrated that astigmatic outcomes in KLEx can be improved using approaches such as intraoperative manual cyclotorsion compensation or cross‐axis alignment [5–7]. Additional methods, such as stringent head positioning, advanced image‐guided surgical systems, and iris registration, have been evaluated to reduce cyclotorsional misalignment [8–11]. However, accurately quantifying and compensating for intraoperative cyclotorsion during KLEx remains challenging. Recent advances in the VisuMax 800 platform offer improved imaging and workflow efficiencies; however, cyclotorsion management remains a key limitation [12–14].

During the postoperative follow‐up of KLEx, notable deviations in the position of the corneal microincision were observed in certain patients. The direction and magnitude of this microincision cyclotorsion may reflect underlying ocular cyclotorsion and thus contribute to misalignment in astigmatic axis correction. Currently, there are few reports evaluating ocular cyclotorsion by analyzing positional changes of the corneal incision during KLEx.

Our study aimed to use MATLAB software to design a method for automatically identifying corneal microincisions, assessing the direction and extent of corneal microincision cyclotorsion during KLEx and evaluating its impact on postoperative astigmatic correction using a vector analysis approach.

## 2. Materials and Methods

### 2.1. Patients

This retrospective study included 92 patients who underwent KLEx at Jinan Mingshui Eye Hospital between January 2024 and March 2025. The study was conducted in accordance with the principles of the Declaration of Helsinki and approved by the Medical Ethics Committee of Jinan Mingshui Eye Hospital. All the participants provided written informed consent.

The inclusion criteria were ages between 17 and 40 years, spherical equivalent (SE) change ≦ 0.50 D in the past 1 year, best corrected visual acuity (BCVA) ≧ 0.8, clear corneas without scarring and with normal morphology, and central corneal thickness (CCT) > 480 μm. The exclusion criteria were strabismus, nystagmus, or any condition affecting the accuracy of ocular cyclotorsion measurements, active ocular diseases, ocular trauma, or previous ocular surgeries.

### 2.2. Preoperative and Postoperative Assessment

All patients underwent preoperative examinations, including uncorrected visual acuity (UCVA), BCVA, refraction, intraocular pressure, slit‐lamp, and fundus examinations. Corneal parameters were measured using a Pentacam anterior segment analysis system. CCT was performed using anterior‐segment optical coherence tomography.

Corneal images were captured at 1–3 months postoperatively using the Haag‐Streit BX 900 illumination system (Haag‐Streit, Switzerland) with an EyeSuite digital camera module. All imaging procedures were performed by experienced operators under natural indoor lighting conditions. A crosslinked laser pen was used for head‐position alignment. The slit‐lamp beam width was set to 3 mm with a light intensity of 60%, and the slit lamp was positioned at a 45 temporal angle to ensure clear imaging of the incision. Patients with unclear incisions or significant incision‐edge opacities were excluded.

**FIGURE 1:**
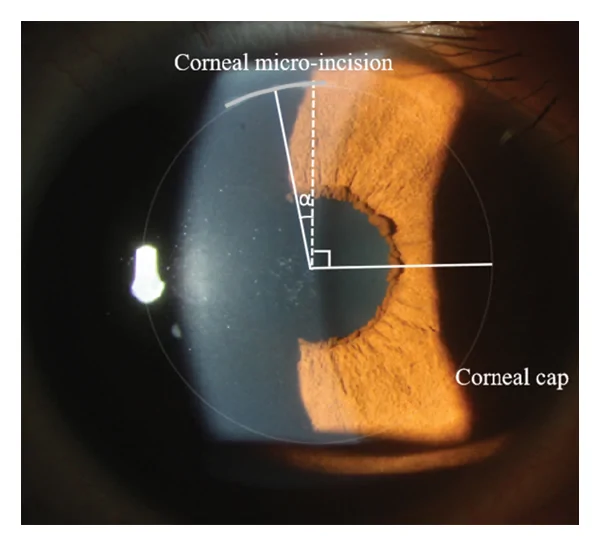
Schematic diagram of the corneal microincision cyclotorsion after KLEx (right eye, *α* angle indicating the cyclotorsion of the corneal microincision).

The corneal microincision angle was measured by fitting a 7.5‐mm diameter circle (corresponding to the KLEx cap diameter) to the curvature of the 2‐mm long microincision arc; the angle between the line connecting the midpoint of the incision arc to the center of the fitted circle and the horizontal axis (0° position of the Cartesian coordinate system) was defined as the incision angle (Figure 1). If cyclotorsion did not occur, the angle was set to 90°. When the angle was > 90°, the right eye incision was considered to be externally rotated, and the left eye incision was considered to be internally rotated. The difference between the measured angle and 90° was defined as the corneal microincision cyclotorsion (*α* angle).

Three measurements were performed for each eye to ensure accuracy. The median value of three measurements, with a deviation of no more than 0.1°, was used as the final degree of cyclotorsion. Image recognition software designed in MATLAB was used to calculate the angle. The steps were as follows: (1) reading and displaying the surgical image containing the incision; (2) using the ginput function to extract incision data; (3) fitting the data to a 7.5‐mm diameter, 2‐mm circular arc; (4) calculating the angular deviation based on the center coordinates of the circle and incision center; and (5) determining the cyclotorsion direction based on the eye side.

### 2.3. Surgical Procedure

All the surgeries were performed by a single experienced surgeon. A VisuMax 500 fs laser system was used for KLEx, with the laser energy set at 135 nJ. The corneal cap diameter was 7.5 mm, cap thickness was 120 μm, and the optical zone diameter ranged from 6.3 to 6.6 mm. The incision was positioned at 90° with a 2‐mm incision length. Prior to surgery, a cross‐line positioning technique was used to ensure accurate head positioning. The patients were instructed to fixate on a steady light with both eyes open, and the laser‐scanning procedure was initiated after aligning the corneal vertex with the center. The lens was removed after completion of the laser scan. Postoperative care included the use of the following eye drops: 0.5% levofloxacin four times daily for 1 week, 0.1% fluorometholone four times daily with weekly tapering for 4 weeks, and 0.1% sodium hyaluronate four times daily for 1–3 months.

### 2.4. Vector Analysis of Astigmatism

Astigmatism correction outcomes were evaluated using alpine vector analysis method [15]. We computed the following parameters at 1 week, 1 month, and 3 months postoperatively: target‐induced astigmatism (TIA), surgically induced astigmatism (SIA), correction index (CI), angle of error (AE), difference vector (DV), index of success (IOS), flattening index (FI), flattening effect (FE), and magnitude of error (ME).

### 2.5. Statistical Analysis

Statistical analyses were performed using IBM SPSS Version 25.0. Normality of the data distribution was assessed using the Kolmogorov–Smirnov test. The absolute value of cyclotorsion was used to represent the magnitude of ocular rotation irrespective of the rotational direction. Paired *t*‐tests were used to compare cyclotorsion between the right and left eyes, whereas independent sample *t*‐tests were used to compare parameters between different groups. Pearson’s correlation analysis was used to evaluate the relationship between cyclotorsion and other clinical variables. Repeated‐measures analysis of variance (ANOVA) was used to compare the vector analysis parameters at different postoperative time points. A *p* value of < 0.05 was considered statistically significant.

## 3. Results

### 3.1. Characteristics of Corneal Microincision Cyclotorsion

A total of 92 patients (184 eyes) were included in this study. The mean absolute microincision cyclotorsion angle was 4.19° ± 2.92°(range: 0°–12.43°) for right eyes and 4.53° ± 3.36°(range: 0°–13.74°) for left eyes. No statistically significant difference was found between the two eyes (*t* = −0.98, *p* = 0.33). Among the right eyes, the distribution of the absolute cyclotorsion angle was as follows: < 5° in 62 eyes (67.39%), 5–10° in 25 eyes (27.17%), and > 10° in 5 eyes (5.43%).

Based on the direction of corneal microincision cyclotorsion, among the right eyes, 74 (80.43%) exhibited outward cyclotorsion of the incision, 13 (14.13%) showed inward cyclotorsion, and 5 (5.43%) showed no detectable cyclotorsion. Among the left eyes, 75 (81.52%) exhibited outward cyclotorsion, 14 (15.22%) showed inward cyclotorsion, and 3 (3.26%) showed no cyclotorsion.

### 3.2. Astigmatic Vector Analysis Over Time

**FIGURE 2:**
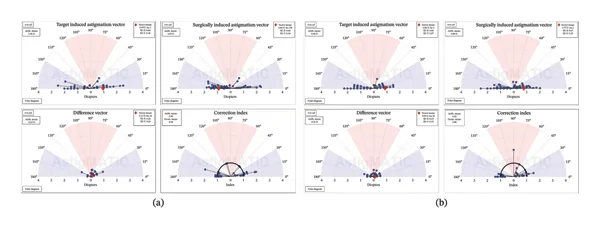
Astigmatic outcomes (alpin criteria) for (a) low cyclotorsion and (b) high cyclotorsion groups.

Vector analysis of astigmatism correction was performed at 1 week, 1 month, and 3 months postoperatively in all 92 eyes. No statistically significant differences were observed among the three time points for any vector component (*p* > 0.05) (Figure 2 and Table 1).

**TABLE 1:** Vector analysis of astigmatic outcomes in total (*n* = 92) eyes following KLEx surgery at different postoperative time points.

Parameter	1‐week postoperative	1‐month postoperative	3‐month postoperative	*F*	*p*
SIA (D)	0.87 ± 0.53	0.86 ± 0.50	0.84 ± 0.50	0.885	0.415
DV (D)	0.24 ± 0.22	0.23 ± 0.23	0.23 ± 0.21	0.294	0.746
CI	1.05 ± 0.79	1.00 ± 0.36	0.97 ± 0.35	0.733	0.482
|AE| (°)	0.10 ± 0.16	0.11 ± 0.23	0.13 ± 0.25	1.198	0.304
IOS	0.39 ± 0.74	0.33 ± 0.42	0.34 ± 0.44	0.640	0.528
FE	0.85 ± 0.53	0.83 ± 0.52	0.81 ± 0.52	1.496	0.227
FI	1.01 ± 0.79	0.92 ± 0.45	0.88 ± 0.46	1.332	0.267
ME (D)	−0.02 ± 0.28	−0.04 ± 0.26	−0.06 ± 0.23	0.885	0.415

Abbreviations: AE = angle of error, CI = correction index, DV = difference vector, FE = flattening effect, FI = flattening index, IOS = index of success, ME = magnitude of error, SIA = surgically induced astigmatism.

The subsequent analyses focused on the right eye. No significant correlations were found between the cyclotorsion magnitude and any of the evaluated preoperative parameters, including age, SE, CCT, mean keratometry (*K*_*m*_), and *Q* value (all *p* > 0.05) (Table 2).

**TABLE 2:** Correlations between the postoperative microincision cyclotorsion and preoperative parameters.

Parameters	Age	SE	CCT	*K*_*m*_	*Q* value
*r*	0.015	−0.072	0.064	0.021	−0.194
*p*	0.889	0.497	0.546	0.843	0.064

*Note:* *K*_*m*_ = mean corneal curvature.

Abbreviations: CCT = central corneal thickness, SE = spherical equivalent.

### 3.3. Comparison of Astigmatism Correction in Low vs. High Cyclotorsion Groups

Patients were stratified into two groups based on cyclotorsion magnitude: A low‐cyclotorsion group (≤ 4°) and a high‐cyclotorsion group (> 4°), with 47 and 45 eyes in each group, respectively. Table 3 shows the baseline characteristics of the two groups. At 3 months postoperatively, no significant differences were observed between the two groups in any of the astigmatic vector parameters (*p* > 0.05). The correction index was 1.04° ± 0.40° in the low‐cyclotorsion group, whereas that in the high‐cyclotorsion group was 0.93° ± 0.32°; however, this difference was not significant (Table 4).

**TABLE 3:** Preoperative characteristics of eyes in the low‐cyclotorsion group and high‐cyclotorsion group.

Parameter	Low‐cyclotorsion group (*n* = 47)	High‐cyclotorsion group (*n* = 45)	*p*
Age (y)	21.11 ± 3.61	21.29 ± 4.08	0.821
CDVA‐logMAR	0 (0, 0.1)	0 (0, 0.1)	0.424
Sphere (D)	−4.16 ± 1.37	−4.40 ± 1.51	0.415
Cylinder (D)	−1.06 ± 0.60	−0.96 ± 0.54	0.416
SE (D)	−4.69 ± 1.45	−4.88 ± 1.57	0.533
CCT (μm)	536.57 ± 28.01	534.91 ± 28.35	0.778
*K*_*m*_ (D)	42.90 ± 1.19	42.84 ± 1.41	0.826

*Note:* CDVA = corrected visual acuity; *K*_*m*_ = mean corneal curvature.

Abbreviations: CCT = central corneal thickness, SE = spherical equivalent.

**TABLE 4:** Subgroup analysis of astigmatic correction based on cyclotorsion magnitude 3 months after KLEx.

Parameter	Low‐cyclotorsion group	High‐cyclotorsion group	*t*	*p*
TIA (D)	0.94 ± 0.55	0.85 ± 0.47	0.838	0.404
SIA (D)	0.90 ± 0.50	0.78 ± 0.50	1.086	0.281
DV (D)	0.24 ± 0.21	0.22 ± 0.20	0.672	0.503
CI	1.02 ± 0.36	0.93 ± 0.32	1.232	0.221
|AE| (°)	0.12 ± 0.19	0.14 ± 0.31	−0.301	0.764
IOS	0.35 ± 0.44	0.33 ± 0.45	0.233	0.816
FE	0.86 ± 0.51	0.75 ± 0.54	1.012	0.314
FI	0.93 ± 0.42	0.83 ± 0.49	1.032	0.305
ME (D)	−0.05 ± 0.24	−0.07 ± 0.23	0.475	0.636

Abbreviations: AE = angle of error, CI = correction index, DV = difference vector, FE = flattening effect, FI = flattening index, IOS = index of success, ME = magnitude of error, SIA = surgically induced astigmatism, TIA = target‐induced astigmatism.

## 4. Discussion

Intraoperative ocular cyclotorsion during KLEx is primarily assessed using manual corneal‐marking techniques or eye‐tracking systems integrated into excimer laser platforms. Song et al. [16] recently described a novel approach using ImageJ image analysis software to assess ocular cyclotorsion after KLEx. However, a major limitation of this approach is the requirement for manual identification of the incision endpoint, which introduces observer‐dependent variability and reduces measurement efficiency. Our present study developed an automated image recognition program using MATLAB, which enables automatic identification of corneal microincisions following KLEx and quantification of incision cyclotorsion angle and direction. This approach provides an objective measure of ocular cyclotorsion that is independent of operator marking and avoids the subjectivity of observer‐dependent marking. The assessment of cyclotorsion in refractive surgery encompasses various techniques, from traditional preoperative manual limbal marking to modern excimer laser platforms with integrated iris‐registration trackers [2–5, 11]. The manual quantification method described by Song et al., which measures corneal incision deviation on slit‐lamp photographs using Image J, currently represents the key reference for indirect, incision‐based cyclotorsion assessment [16]. Our MATLAB‐based innovative method builds upon this validated principle through automation. It eliminates the manual steps of image orientation and landmark selection, reduces processing time to seconds per eye, and thereby addresses the core limitations associated with manual techniques.

In the present study, the mean absolute microincision cyclotorsion angle following KLEx was measured to be 4.19° ± 2.92° (range: 0°–12.43°) in the right eyes and 4.53° ± 3.36° (range: 0°–13.74°) in the left eyes, which is consistent with the findings reported by Zhao et al. [17] and Terauchi et al. [18]. Using our automated tool, quantitative analysis revealed that the majority of eyes demonstrated slight excyclotorsion at the incision site. These findings suggest a predominant tendency for incyclotorsion during surgery. To minimize deviations caused by head tilt, a cross‐axis alignment method was employed both preoperatively and intraoperatively. Previous studies have demonstrated that this method can effectively reduce axial misalignment and ensure optimal postoperative astigmatic correction [7, 19]. In addition to patient cooperation, the intraoperative cyclotorsion observed during KLEx surgery may be related to surgical draping and an accommodative convergence response [20]. The latter could be induced by blurred vision of the fixation target during laser scanning, which may trigger an accommodative effort, and because accommodation is coupled with convergence, a subsequent cyclotorsion may occur. However, direct evidence in KLEx is currently lacking, and this remains a speculative mechanism.

In the analysis of preoperative factors influencing ocular cyclotorsion, most existing studies have focused on the determinants of static and dynamic cyclotorsion in excimer laser procedures [11, 17, 21, 22], whereas reports on factors affecting intraoperative cyclotorsion during KLEx remain limited. Song et al. [16] reported that a smaller preoperative sphere was associated with a higher amount of cyclotorsion. In contrast, we found no statistically significant correlation between the magnitude of incision cyclotorsion and any preoperative parameter. This discrepancy may reflect differences in cohort characteristics, such as the range of preoperative sphere or the proportion of low‐to‐moderate astigmatism, rather than methodological differences, as both studies used an incision‐based measurement.

Some studies have already demonstrated significant results when using cyclotorsion compensation to correct astigmatism [23, 24], whereas others have found no effect on astigmatism correction or visual quality improvement [6, 25, 26]. This may be related to factors such as positioning methods, cyclotorsion compensation methods, astigmatism magnitude, and sample size differences adopted in various studies. When eyes were stratified into low or high cyclotorsion groups, we observed no significant differences in any of the astigmatic vector analysis parameters. This finding is consistent with previous reports suggesting that modest intraoperative cyclotorsion has a limited impact on the KLEx results [2, 6, 26].

A recent systematic review and meta‐analysis reported that cyclotorsion compensation led to a statistically significant reduction in postoperative residual astigmatism. However, that analysis also found no significant differences in astigmatism vector indices, such as TIA, CI, IOS, or the magnitude of error [23]. Similarly, a randomized contralateral eye trial found no difference in visual or refractive outcomes between KLEx eyes with and without manual cyclotorsion compensation [25]. Our results suggest that small degrees of ocular cyclotorsion may be well tolerated, especially in cases of low‐to‐moderate astigmatism, underscoring the need to define a threshold at which cyclotorsion becomes clinically significant. If cyclotorsion exceeds a certain angle, targeted compensation may yield benefits.

According to recent studies, the second‐generation femtosecond laser system VisuMax 800 can dynamically compensate for ocular cyclotorsion during KLEx by establishing real‐time docking and continuously monitoring cyclotorsional eye movements throughout the laser procedure [12]. In some studies, this system has demonstrated promising refractive outcomes with improved precision. However, other studies found that the addition of cyclotorsion compensation features yields limited visual benefits in certain patient groups [13, 14]. These mixed findings suggest that the current cyclotorsion compensation strategies require further optimization. Further clinical research is needed to understand the individual variations in ocular cyclotorsion and their influence on surgical outcomes, which may ultimately inform the development of more personalized and effective compensation algorithms.

This study has several limitations. First, the analysis was conducted at a single center by a single surgical team, which may limit the generalizability of the findings. Second, the study cohort consisted predominantly of eyes with low to moderate astigmatism (mean cylinder: −1.01 ± 0.57 D; range: −0.25 D–−2.50 D), with limited inclusion of patients requiring high cylindrical correction. Consequently, the applicability of our results to populations with higher astigmatism may be restricted, and future studies encompassing a broader range of refractive errors are warranted. Third, the cyclotorsion measurements depended on the quality and orientation of postoperative slit‐lamp photographs; imaging tilt or misalignment could introduce bias in the estimates. Specifically, the performance of our automated MATLAB method relies on high‐quality images with well‐defined incision margins. Moreover, the algorithm parameters were optimized for the specific imaging device and scan protocol employed here, and its generalizability to other platforms requires further validation and potential recalibration. Finally, the four cutoff used for subgroup analysis was chosen based on convention; the clinically meaningful threshold for cyclotorsion remains to be established. As an observational study, no intervention was applied to correct the measured cyclotorsion; therefore, we cannot directly evaluate the effect of a compensation strategy on refractive outcomes.

In summary, the corneal microincision cyclotorsion measurement method designed in this study is suitable for the evaluation of ocular cyclotorsion. This measurement method is easy to implement and is based on actual surgical outcomes, providing clinical guidance for research on ocular cyclotorsion and compensation. Through the measurement of corneal microincision cyclotorsion, we found that cyclotorsion had a negligible impact on astigmatism correction. These findings suggest that routine compensation for minor cyclotorsion may be unnecessary in standard KLEx procedures although further studies are warranted to determine its value in highly astigmatic or complex cases.

## Author Contributions

Jie Hou contributed to the design and interpretation of data and wrote the manuscript. Feng Rao contributed to the topic selection and designed the measurement software. Yulin Lei contributed to the revision of the manuscript. Jing Zhang and Yahui Dong contributed to the data collection and provided supportive contributions.

## Funding

This work was supported by the Science and Technology Development Program Project of Jinan Municipal Health Commission (2024306016) and the Research Project of Shandong School Health Association (SDWS2026019).

## Conflicts of Interest

The authors declare no conflicts of interest.

## Data Availability

The data that support the findings of this study are available from the corresponding author upon reasonable request.
